# Interaction Between Frailty and Renal Function in Patients with Heart Failure

**DOI:** 10.3390/life16010045

**Published:** 2025-12-26

**Authors:** Ángela Rodríguez-Eguren, José Jesús Broseta, Lydia Izquierdo, Joan Llevadot-Sesmilo, Eduard Solé Gonzalez, María Ángeles Castel, Juan José Rodriguez, Elena Cuadrado-Payán, Diana Rodriguez-Espinosa, Elena Sandoval, Aleix Cases, Francisco Maduell, Ana García-Álvarez, Marta Farrero, Pedro Caravaca-Pérez

**Affiliations:** 1 Heart Failure and Heart Transplant Unit, Institut Clínic Cardiovascular (ICCV), Hospital Clínic, Universitat de Barcelona, 08007 Barcelona, Spain; arodrigueze@clinic.cat (Á.R.-E.); lyizquie@clinic.cat (L.I.); jllevadot@clinic.cat (J.L.-S.); edsole@clinic.cat (E.S.G.); macastel@clinic.cat (M.Á.C.); anagarci@clinic.cat (A.G.-Á.); mfarrero@clinic.cat (M.F.); 2 Institut d’Investigacions Biomèdiques August Pi I Sunyer (IDIBAPS), 08036 Barcelona, Spain; jjbroseta@clinic.cat (J.J.B.); ecuadrado@clinic.cat (E.C.-P.); dmrodriguez@clinic.cat (D.R.-E.); esandova@clinic.cat (E.S.); acases@clinic.cat (A.C.); fmaduell@clinic.cat (F.M.); 3 Nephrology and Renal Transplantation, Hospital Clínic de Barcelona, 08036 Barcelona, Spain; 4 Laboratori Experimental de Nefrologia i Trasplantament (LENIT), 08036 Barcelona, Spain; 5 Department of Medicine, Universitat de Barcelona, 08007 Barcelona, Spain; 6 Centro de Investigación Biomédica en Red, Enfermedades Cardiovasculares (CIBERCV), 28029 Madrid, Spain; 7 Cardiovascular Surgery Department, Hospital Clinic de Barcelona, 08036 Barcelona, Spain; 8 Centro Nacional de Investigaciones Cardiovasculares (CNIC), 28029 Madrid, Spain

**Keywords:** heart failure, chronic kidney disease, frailty

## Abstract

**Background.** Frailty is highly prevalent among patients with heart failure (HF) and is associated with adverse clinical outcomes. Chronic kidney disease (CKD) frequently coexists with HF and may further increase risk. However, the clinical profile linking frailty and CKD remains insufficiently characterized. This study aimed to determine the prevalence and clinical correlates of frailty in outpatients with HF and to assess whether its prognostic significance varies across CKD severity. **Methods.** A prospective, observational cohort of HF outpatients was enrolled. Frailty was defined according to Fried’s phenotype (≥3 criteria). Factors associated with frailty were identified using logistic regression. The primary endpoint was a composite of all-cause mortality or HF hospitalization over one year. Cox proportional hazards models were used to evaluate associations between frailty and outcomes and to test its interaction with CKD. **Results.** A total of 459 HF outpatients (median age 75 [IQR 68–82] years; 72% men) were included. Frailty was present in 39.9% of patients and increased progressively with worsening renal function—from 14% in those with eGFR >60 to 38% in eGFR 30–60 and 48% in eGFR <30 mL/min/1.73 m^2^ (*p* < 0.001). In multivariate analysis, older age, prior stroke, higher CA125 levels, and lower eGFR were independently associated with frailty. Frail patients had a higher risk of all-cause death or HF hospitalization (adjusted HR 2.09; 95% CI 1.22–3.58; *p* = 0.007), with an amplified effect among those with advanced CKD (HR 5.02; 95% CI 2.46–10.22; *p* < 0.001). **Conclusions.** In HF outpatients, frailty is common and closely linked to renal dysfunction. Its coexistence with advanced CKD identifies a subgroup at the highest risk of adverse outcomes. Combined assessment of frailty and renal function may enhance prognostic precision and guide more individualized therapeutic strategies.

## 1. Introduction

Frailty is a multifactorial geriatric syndrome characterized by diminished physiological reserve and impaired function across multiple organ systems [1]. It compromises the capacity to maintain homeostasis and increases vulnerability during both chronic and acute stressors [1]. Among patients with HF, frailty is highly prevalent and independently associated with increased risks of hospitalization and mortality across the spectrum of left ventricular ejection fraction (LVEF) [2,3,4].

CKD is also common in HF and contributes to adverse outcomes through the cardiorenal syndrome [5]. Shared pathophysiological mechanisms—including systemic inflammation, endothelial dysfunction, congestion, and metabolic disturbances—may further exacerbate clinical deterioration [6].

A substantial proportion of HF patients exhibit both frailty and renal impairment. Nonetheless, within the HF continuum, these conditions are typically assessed separately, limiting comprehensive risk evaluation. Given their shared mechanisms and frequent coexistence, integrating assessments of frailty and renal dysfunction may provide incremental prognostic value. We hypothesized that the coexistence of frailty and CKD delineates a distinct, high-risk HF phenotype. The primary objective of this study was to examine the association between renal dysfunction and frailty prevalence and to determine the prognostic implications of their coexistence. Secondary objectives were to identify clinical predictors of frailty and explore their relationship with renal function.

## 2. Materials and Methods

### 2.1. Study Design and Participants

A prospective, observational, single-center study was conducted at Hospital Clínic de Barcelona between 1 January 2022, and 31 December 2024, to investigate the association between frailty and renal function in HF patients. Consecutive outpatients with confirmed HF were enrolled.

Inclusion criteria were age ≥65 years, a diagnosis of chronic HF according to European Society of Cardiology (ESC) criteria [7], clinical stability at assessment, available renal data for eGFR calculation, and a complete frailty evaluation at the index visit. Exclusion criteria were severe cognitive impairment precluding reliable frailty assessment (Pfeiffer score >4 errors), active malignancy with life expectancy <1 year, acute HF decompensation at assessment, or incomplete frailty data (≥3 missing domains precluding Fried classification). Patients for whom adequate follow-up was not expected from the outset were also excluded.

Clinical evaluations were conducted by HF-specialized cardiologists following guideline-directed therapy [7]. Patients with advanced CKD were additionally reviewed in a multidisciplinary cardiorenal clinic. The study protocol was approved by the institutional ethics committee (HCB/2022/1122), and all participants provided written informed consent.

### 2.2. Data Collection

Baseline demographic, clinical, echocardiographic, and laboratory data were prospectively retrieved from electronic records. Laboratory parameters included complete blood count, renal and hepatic panels, fasting glucose, lipid profile, N-terminal-pro–B-type natriuretic peptide (NT-proBNP), and cancer antigen 125 (CA125). A spot urine sample was obtained for albumin-to-creatinine ratio and urinary electrolytes. Frailty and renal assessments were performed during the same encounter or within 30 days. Echocardiographic parameters, including LVEF, were derived from the most recent transthoracic echocardiogram within 12 months.

### 2.3. Baseline Variables

Frailty was assessed by the Fried physical phenotype, encompassing five domains [8,9]. Weakness was quantified by handgrip strength measured with a handheld dynamometer, applying sex- and body mass index (BMI)-specific cut-off values consistent with the original Fried criteria (≤29, ≤30, ≤30, and ≤32 kg in men and ≤17, ≤17.3, ≤18, and ≤21 kg in women across increasing BMI categories). Slowness was assessed using a standardized 4 m gait speed test, with height- and sex-adjusted thresholds (≥7 s for men ≤173 cm and women ≤159 cm; ≥6 s for taller individuals). Unintentional weight loss was defined as a self-reported reduction of ≥4.5 kg or ≥5% of body weight over the preceding year. Exhaustion was identified through self-reported responses to validated items assessing fatigue and diminished energy during daily activities. Low physical activity was defined on the basis of self-reported reduction in habitual activity, using standardized clinical questioning. Each criterion was scored as 1 if present and 0 if absent, yielding a frailty score ranging from 0 to 5. Participants meeting ≥ 3 criteria were classified as frail, whereas those fulfilling 0–2 criteria were categorized as nonfrail or prefrail and served as the reference group.

Comprehensive geriatric assessment included functional dependence (Barthel Index) [10], comorbidity burden (Charlson Comorbidity Index) [11], nutritional status (Mini Nutritional Assessment–Short Form, MNA-SF) [12], and cognitive function (Pfeiffer Short Portable Mental Status Questionnaire) [13]. eGFR was calculated with the Chronic Kidney Disease Epidemiology Collaboration (CKD-EPI) equation and classified per Kidney Disease Improving Global Outcomes (KDIGO) criteria into ≥60 mL/min/1.73 m^2^, 30–59 mL/min/1.73 m^2^, and <30 mL/min/1.73 m^2^, the latter category including patients receiving chronic dialysis. Albuminuria was categorized as A1 (<30 mg/g), A2 (30–300 mg/g), or A3 (>300 mg/g). For descriptive purposes, the eGFR and albuminuria categories were cross-tabulated to derive KDIGO heatmap risk strata (from low to very high risk). These combined renal parameters were used to contextualize frailty burden along the continuum of cardiorenal risk.

LVEF was measured using the biplane Simpson’s method and categorized as preserved (≥50%), mildly reduced (40–49%), or reduced (≤40%) [7].

Missing data were handled using a complete case analysis. No imputation procedures were applied. To enhance transparency, the number of observations available was explicitly reported in tables or figures for variables with substantial missingness.

### 2.4. Follow-Up and Outcomes

Follow-up data were collected from electronic records and scheduled visits. Clinical events were adjudicated by physicians blinded to frailty status and reviewed by a senior cardiologist. The primary endpoint was a composite of all-cause mortality or HF hospitalization within one year.

### 2.5. Ethical Considerations

The study was conducted in accordance with the Declaration of Helsinki and Good Clinical Practice guidelines. The protocol was approved by the Institutional Ethics Committee of Hospital Clínic de Barcelona. Data were anonymized and processed in compliance with European Union General Data Protection Regulation requirements to ensure confidentiality. Personal identifiers were removed from the analytical dataset, and only coded study identifiers were used for statistical analyses.

### 2.6. Statistical Analysis

Continuous variables were summarized as median (interquartile range [IQR]) and compared using the Mann–Whitney U test. Categorical variables were presented as absolute numbers and percentages and compared by χ^2^ test or Fisher’s exact test, as appropriate. Prior to multivariable modeling, the linearity of continuous predictors with the logit was examined; variables with non-linear relationships were log-transformed (natural logarithm) for both univariate and multivariate analyses. These included CA125, NT-proBNP, creatine kinase, and alkaline phosphatase.

Associations with frailty were initially evaluated using univariate logistic regression. Variables with *p* < 0.10 in univariate analyses, together with covariates considered clinically relevant, were entered into a multivariate logistic regression model using a backward stepwise selection procedure. Model discrimination and calibration were assessed by the area under the receiver operating characteristic curve (AUC/ROC), the Hosmer–Lemeshow goodness-of-fit test, and model specification using a link test.

Time-to-event analyses were performed using Kaplan–Meier survival estimates, log-rank tests, and Cox proportional hazards regression models. A similar backward stepwise approach was applied to derive the final multivariable Cox model. To investigate whether the association between frailty and clinical outcomes differed according to renal function, an interaction term between frailty status and eGFR category was incorporated into the multivariable Cox model. eGFR was stratified into three predefined categories (>60, 30–60, and <30 mL/min/1.73 m^2^ or dialysis). The statistical significance of the interaction was evaluated using the Wald test applied to the frailty × eGFR category cross-product term. Additionally, stratified analyses were conducted to estimate hazard ratios for frailty within each eGFR category.

A two-sided *p*-value < 0.05 was considered indicative of statistical significance. All statistical analyses were conducted using Stata version 19 (StataCorp, College Station, TX, USA).

## 3. Results

**Figure 1:**
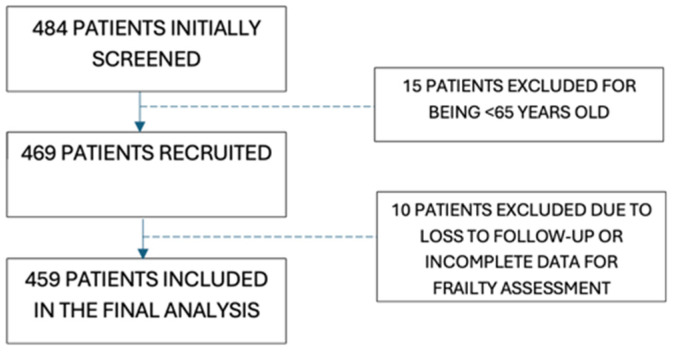
Flow diagram of patient selection and inclusion. A total of 484 patients with chronic heart failure were screened; 15 were excluded for being <65 years of age, leaving 469 participants recruited. Of these, 10 were excluded because clinical follow-up could not be ensured or because frailty could not be classified due to insufficient data (missing ≥3 frailty domains). The final study cohort comprised 459 patients who completed frailty assessment and were included in the survival analyses.

A total of 484 patients were screened. After excluding patients younger than 65 years and those with incomplete frailty data, 459 patients were included in the final analysis (Figure 1).

**Figure 2:**
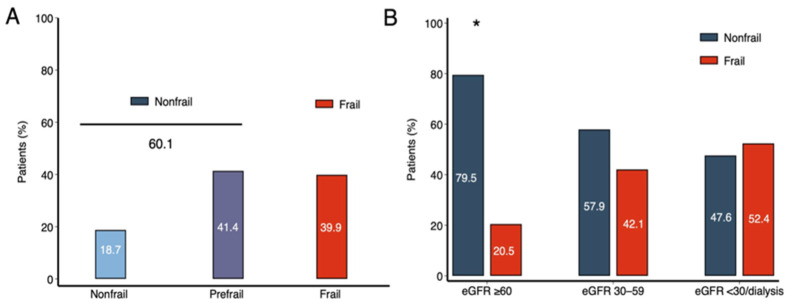
(**A**) Distribution of frailty in the overall cohort. (**B**) Frailty prevalence across renal function categories. (**A**) Prevalence of frailty according to the Fried phenotype in the full cohort. Data are expressed as the percentage of the total population. (**B**) Distribution of frailty status across renal function strata defined by estimated glomerular filtration rate (eGFR): ≥60 mL/min/1.73 m^2^ (n = 127), 30–59 mL/min/1.73 m^2^ (n = 164), and <30 mL/min/1.73 m^2^ or dialysis (n = 168). Data are expressed as percentages. Frailty prevalence increased progressively with worsening renal function (*p* < 0.001, χ^2^ test). * Indicates statistically significant differences across eGFR categories. Abbreviation: eGFR, estimated glomerular filtration rate.

Table 1 summarizes baseline characteristics. Median age was 75 years (IQR 68–82), and 72% were men. Median left ventricular ejection fraction (LVEF) was 40% (IQR 30–55); 37% of patients had preserved (≥50%), 18% mildly reduced (40–49%), and 45% reduced LVEF (≤40%). Overall, 39.9% of the cohort met frailty criteria (≥3 points), while 41.4% were prefrail and 18.7% nonfrail, yielding a combined prevalence of 60.1% for prefrail/nonfrail status (Figure 2A). Supplementary Table S1 shows the distribution of frailty domains in frail and non-frail patients.

### 3.1. Frailty Across Stages of Renal Dysfunction

Median eGFR was 38 mL/min/1.73 m^2^ (IQR 24–63); 28% of patients had eGFR ≥ 60 mL/min/1.73 m^2^, 36% between 30 and 59 mL/min/1.73 m^2^, and 36% < 30 mL/min/1.73 m^2^ (including dialysis). Frail patients had significantly lower eGFR (median 30 vs. 48 mL/min/1.73 m^2^; *p* < 0.001). As illustrated in Figure 2B, the proportion of frail individuals increased progressively with worsening renal function: 26 (20.5%) for eGFR > 60, 69 (42.1%) for eGFR 30–60, and 88 (52.4%) for eGFR < 30 mL/min/1.73 m^2^ (*p* < 0.001).

**Figure 3:**
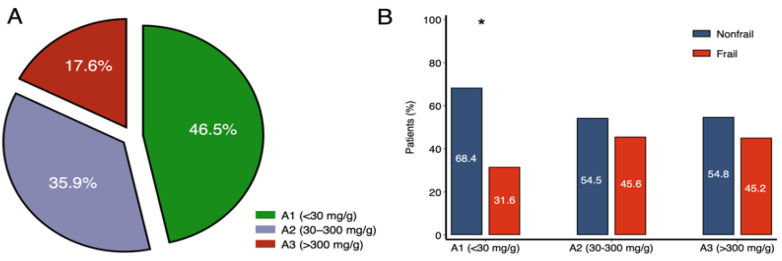
(**A**) Distribution of albuminuria categories in the overall cohort. (**B**) Distribution of frailty status within each albuminuria category. (**A**) Pie chart showing the distribution of KDIGO albuminuria categories: A1 (<30 mg/g, green; n = 193), A2 (30–300 mg/g, purple; n = 149), and A3 (>300 mg/g, red; n = 73). Values represent the percentage contribution of each category to the total population. (**B**) Frailty prevalence across albuminuria categories. Bars represent the percentage of nonfrail (blue) and frail (red) patients within each KDIGO category (A1: nonfrail 132 [68.4%], frail 61 [31.6%]; A2: nonfrail 81 [54.5%], frail 68 [45.6%]; A3: nonfrail 40 [54.8%], frail 33 [45.2%]). The prevalence of nonfrail patients decreased with increasing albuminuria severity (*p* = 0.015, χ^2^ test). * Indicates statistically significant differences across albuminuria categories. Abbreviations: KDIGO, Kidney Disease: Improving Global Outcomes; A1, normal to mildly increased albuminuria (<30 mg/g); A2, moderately increased albuminuria (30–300 mg/g); A3, severely increased albuminuria (>300 mg/g).

Median UACR was 36 mg/g (IQR 9–175); 46% were classified as A1 (<30 mg/g), 36% as A2 (30–300 mg/g), and 18% as A3 (>300 mg/g). Frailty prevalence differed by albuminuria categories (*p* = 0.015), with the higher proportion of nonfrail individuals in the A1 group (Figure 3).

**Figure 4:**
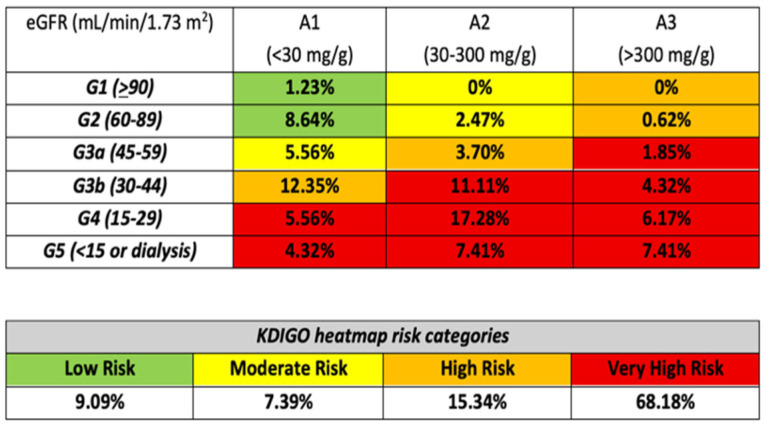
Distribution of frail patients according to KDIGO heatmap risk categories. The upper panel includes only frail patients (n = 162) and displays, for each combination of albuminuria category (A1–A3) and eGFR stage (G1–G5), the percentage of all frail patients falling within that albuminuria/eGFR stratum. The lower panel summarizes the proportion of all frail patients in each KDIGO risk category (low, moderate, high, and very high risk), showing that frail patients cluster predominantly in the high and very high KDIGO risk categories. Color coding follows KDIGO convention: green, low risk; yellow, moderate risk; orange, high risk; red, very high risk. Abbreviations: KDIGO, Kidney Disease: Improving Global Outcomes; A1, albuminuria <30 mg/g; A2, 30–300 mg/g; A3, >300 mg/g; G1–G5, categories of estimated glomerular filtration rate (eGFR; mL/min/1.73 m^2^).

In the overall cohort, according to KDIGO heatmap risk stratification, 81 patients (18%) were classified as low risk, 61 (14%) as moderate risk, 68 (16%) as high risk, and 229 (52%) as very high risk. Figure 4 displays the distribution of frailty across KDIGO risk categories, integrating eGFR and albuminuria stages. Frailty burden increased with greater KDIGO risk, with most frail patients clustering in the very high-risk strata (68%). The highest proportion of frail individuals was observed in CKD stages G3b–G5, particularly in G4–A2 (17%). In contrast, frailty was rare in CKD stage G1 (<1%).

In logistic regression, frailty was associated with older age, female sex, lower eGFR, and higher comorbidity (atrial fibrillation, diabetes, prior stroke) (Table 2). Multivariate analysis confirmed independent associations with age, eGFR, CA125, and stroke (Table 3). Model discrimination was strong (AUC ≈ 0.85).

### 3.2. Frailty, Renal Function, and Clinical Outcomes

**Figure 5:**
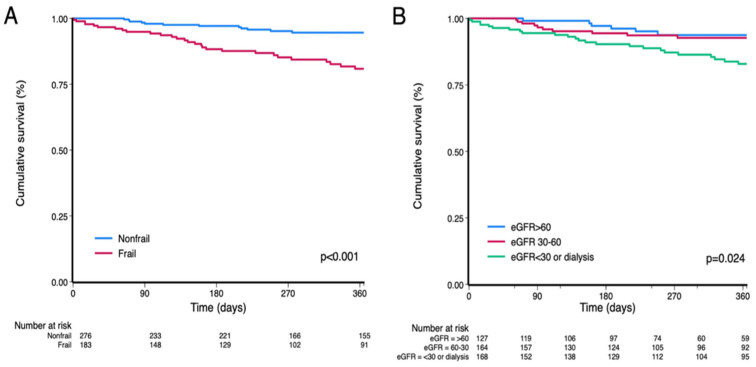
One-year survival for the primary outcome by (**A**) frailty status and (**B**) renal function. Kaplan–Meier curves depicting survival according to (**A**) frailty status (frail vs. nonfrail) and (**B**) renal function strata based on eGFR (≥60, 30–59, and <30 mL/min/1.73 m^2^ or dialysis). Survival probability represents freedom from the primary outcome over 1 year of follow-up. Numbers at risk for each group are shown below the x-axis. Survival was significantly lower in frail than in nonfrail patients (*p* < 0.001, log-rank test) and decreased progressively with worsening renal function (*p* = 0.024, log-rank test). Abbreviation: eGFR, estimated glomerular filtration rate.

Over one year, 52 patients (11%) were hospitalized for HF and 33 (7%) died, yielding a total of 78 composite events (17%). Frail patients experienced significantly higher event rates (*p* < 0.001) (Figure 5A). In the Cox analysis, frailty conferred a 2.81-fold higher risk (95% CI 1.77–4.43; *p* < 0.001). Lower eGFR was independently associated with an increased risk of adverse outcomes (hazard ratio [HR] per 1 mL/min/1.73 m^2^ increase, 0.98; 95% CI 0.97–0.99; *p* = 0.004), and Kaplan–Meier curves demonstrated a graded decline in event-free survival across eGFR categories (*p* = 0.024) (Figure 5B). After adjustment, frailty remained independently associated with the composite outcome (HR 2.09; 95% CI 1.22–3.58; *p* = 0.007; C-statistic = 0.74; Table 3).

**Figure 6:**
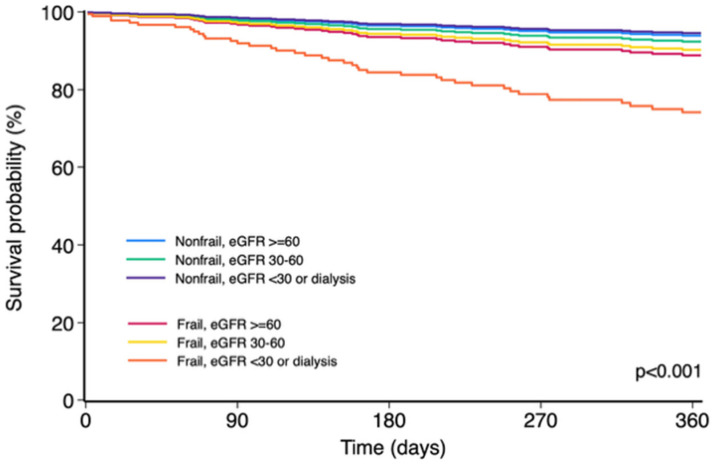
One-year survival estimates according to combined frailty status and renal function. Survival curves are derived from a Cox proportional hazards model including frailty status (frail vs. nonfrail) and renal function categories based on estimated glomerular filtration rate (eGFR ≥60, 30–59, and <30 mL/min/1.73 m^2^ or dialysis), with an interaction term between frailty and renal function (FRAGILIDAD × categoria_fg). The x-axis shows time from baseline to 1-year follow-up (days), and the y-axis shows the model-based estimated probability of remaining free from the primary outcome. Patients who were both frail and had severely reduced eGFR (<30 mL/min/1.73 m^2^ or dialysis) had the poorest prognosis. There was a statistically significant overall difference in survival across the six frailty–eGFR subgroups (*p* < 0.001, Cox model). Abbreviation: eGFR, estimated glomerular filtration rate.

In stratified analyses, renal function appeared to modify the relationship between frailty and outcomes. Frailty conferred a modest, nonsignificant increase in risk among patients with preserved or moderately impaired renal function, but a pronounced and independent increase in risk among those with severe renal dysfunction (HR 5.02; 95% CI 2.46–10.22; *p* < 0.001). Survival curves derived from the Cox model (Figure 6) illustrated a progressively worse prognosis with increasing renal dysfunction, with frail patients with advanced CKD exhibiting the highest event rates. Although a risk gradient was evident, the formal test for interaction between frailty and eGFR categories (>60, 30–60, and <30 mL/min/1.73 m^2^ or dialysis) did not reach statistical significance (*p* = 0.28). Albuminuria was not independently associated with adverse outcomes (*p* = 0.82).

## 4. Discussion

This study examined the interplay between frailty and renal dysfunction in patients with HF, as well as their combined prognostic implications. Four principal findings emerged. First, frailty was highly prevalent in HF and identified a subgroup of patients with greater clinical complexity, higher comorbidity burden, and increased overall risk. Second, a bidirectional relationship was observed between frailty and renal function: frailty was more frequent among individuals with impaired kidney function, while renal dysfunction was more common in those classified as frail. Third, independent determinants of frailty included advanced age, reduced estimated glomerular filtration rate (eGFR), congestion—reflected by elevated CA125 levels—and a history of stroke. Fourth, frail patients with concomitant renal dysfunction experienced a substantially higher incidence of adverse clinical events, with an amplified prognostic effect when both conditions coexisted. Collectively, these findings demonstrate that the coexistence of frailty and renal impairment provides prognostic information beyond that offered by either condition alone, underscoring the added value of incorporating frailty assessment into the evaluation of renal dysfunction in HF.

With the progressive aging of the HF population and improved survival conferred by disease-modifying therapies, the need for refined tools to quantify biological vulnerability has become increasingly evident. In this context, frailty has emerged as a clinically relevant geriatric syndrome and a key prognostic modifier in HF [2,4]. Reported frailty prevalence in HF varies substantially across studies. Meta-analyses have estimated prevalence rates between 38% and 45% in older adults and unselected HF cohorts [14,15], with higher rates generally observed in studies using multidimensional or deficit-accumulation models that classify a broader spectrum of individuals as frail. In contrast, investigations employing the Fried frailty phenotype—the operational framework applied in the present analysis—have typically reported lower prevalence estimates [16]. In our cohort, frailty was identified in 39.9% of patients, consistent with prior data. The Fried phenotype remains the most widely used construct in HF research and has demonstrated incremental prognostic value [16]; however, no frailty instrument has been specifically validated for HF populations.

In this cohort, frail patients displayed a distinct clinical profile characterized by older age, a higher prevalence of atrial fibrillation, and more frequent prior stroke. Biochemically, they exhibited higher NT-proBNP and lower hemoglobin levels, indicative of a more advanced, congestive HF phenotype [17]. Notably, frail patients more often had preserved LVEF (*p* = 0.003), whereas nonfrail patients more frequently presented with reduced LVEF (*p* = 0.012). This observation aligns with prior evidence that frailty is particularly common in HF with preserved ejection fraction (HFpEF), especially among older adults and women with multiple comorbidities such as hypertension, diabetes mellitus, obesity, and CKD [4]. In this setting, frailty tends to cluster with systemic congestion, metabolic disturbances, and a high comorbidity load rather than with isolated systolic dysfunction.

Therapeutically, frail patients were less frequently treated with sacubitril/valsartan, beta-blockers, and mineralocorticoid receptor antagonists and more often received loop diuretics. These patterns may partially reflect the higher proportion of HFpEF, in which the benefits of neurohormonal blockade remain less well established, as well as a more conservative therapeutic approach in frail individuals driven by concerns about drug tolerability, polypharmacy, multimorbidity, or advanced age [18]. Nevertheless, growing evidence indicates that guideline-directed medical therapy (GDMT) remains both efficacious and safe in frail patients and that, given their elevated baseline risk, they may derive greater absolute benefit from optimized pharmacological treatment [4].

### 4.1. Predictors of Frailty

Age emerged as a strong and biologically plausible predictor of frailty, reflecting the cumulative effects of aging-related mechanisms such as immunosenescence, oxidative stress, mitochondrial dysfunction, and progressive loss of physiological reserve [19]. Renal dysfunction was also independently associated with frailty, extending prior observations [20,21,22]. In our cohort, lower eGFR remained a significant predictor of frailty after extensive multivariable adjustment, reinforcing the concept that renal impairment represents a core component of the frailty phenotype rather than a coincidental comorbidity. Although the validity of creatinine-based eGFR in frail or sarcopenic patients has been questioned [23,24], it retained both clinical and prognostic relevance in this study, underscoring its utility as a simple and widely accessible marker of renal function in patients with HF and frailty.

Among circulating biomarkers, CA125 emerged as an independent correlate of frailty, suggesting a role beyond its conventional use as a congestion marker [25]. This association is pathophysiologically plausible, as CA125 reflects venous congestion and systemic metabolic stress, both implicated in the pathogenesis and progression of frailty. Other laboratory parameters, including blood urea nitrogen, hemoglobin, and creatine kinase, were not retained in the multivariable model, likely due to shared pathophysiological pathways and collinearity with more informative variables. The final predictive model demonstrated good discrimination, with an area under the receiver operating characteristic curve of approximately 0.85.

### 4.2. Prognostic Impact of Frailty and Kidney Function

Frailty and renal dysfunction capture distinct yet complementary dimensions of disease severity in HF. Their coexistence defines a high-risk phenotype not fully recognized by either condition alone. Frail patients experienced higher rates of HF hospitalization and all-cause mortality, and this association persisted after multivariable adjustment, confirming frailty as an independent prognostic marker, consistent with previous reports [2,3,4,21,26].

Renal function also exerted a significant impact on survival, as lower eGFR was associated with increased mortality risk. Given its established prognostic role, renal function should be integrated into risk stratification and therapeutic decision-making [27]. Although the interaction test did not reach statistical significance (*p* = 0.28), a consistent and clinically meaningful gradient was observed: patients who were both frail and had advanced CKD experienced the highest event rates. This pattern suggests a synergistic effect on prognosis beyond the contribution of either condition alone. Such synergy likely reflects convergent biological mechanisms—including inflammation, neurohormonal activation, congestion, and metabolic derangements—that collectively amplify vulnerability to decompensation and death.

These findings delineate a distinct, high-risk phenotype: frail HF patients with advanced CKD, consistent with data from cohorts with end-stage renal disease [20]. Clinically, this underscores the importance of systematically integrating frailty assessment into the management of HF patients with renal impairment. This approach may facilitate the identification of individuals at greatest risk who warrant intensified monitoring, individualized therapeutic goals, and early, structured discussions regarding prognosis, goals of care, and treatment intensity.

### 4.3. Clinical Implications and Contribution

This study underscores the high prevalence of frailty among HF patients and its strong interplay with renal dysfunction in determining outcomes. Patients exhibiting both frailty and renal impairment constituted a subgroup with markedly elevated risk. The use of a well-characterized cohort, structured follow-up, and a validated frailty assessment tool supports the internal consistency and translational relevance of these findings.

Identifying this vulnerable subgroup may enable the implementation of targeted interventions with proven benefits, such as structured exercise programs and nutritional support [28,29]. In this high-risk population, intensified clinical surveillance, optimization of guideline-directed therapy, and interventions aimed at mitigating or reversing frailty may help reduce adverse clinical events.

### 4.4. Limitations

This study has several limitations that warrant careful consideration. Its single-center, observational design within a specialized HF clinic may limit external validity and precludes causal inference. Patients referred to a tertiary care program are typically more clinically stable and receive more intensive monitoring, potentially introducing selection bias relative to the broader HF population.

Frailty was assessed exclusively using the Fried physical phenotype and measured only at baseline; thus, temporal changes could not be evaluated. Although extensively validated in HF research, the absence of additional performance-based, cognitive, or multidimensional frailty measures may limit the precision with which frailty severity and domains were characterized. Renal function was estimated using creatinine-based eGFR, which may underestimate dysfunction in sarcopenic individuals; combined or cystatin C–based equations could improve accuracy.

Psychosocial, behavioral, and lifestyle determinants were not systematically captured and may confound or modify the observed associations. The sample size may have limited the detection of modest interaction effects, particularly across KDIGO strata. Event adjudication relied on clinical documentation and electronic health records without uniform biomarker confirmation, potentially leading to misclassification.

These findings require external validation in larger, more heterogeneous cohorts and should be interpreted as hypothesis-generating rather than evidence of causality. Although multivariable models controlled for key confounders—including age, sex, comorbidities, congestion markers, and renal function—residual confounding cannot be fully excluded. Frailty and renal dysfunction often coexist with multimorbidity, polypharmacy, reduced exercise tolerance, and chronic congestion, which may independently worsen outcomes. Therefore, the observed associations should be regarded as robust but not necessarily causal.

In summary, frailty was highly prevalent among patients with HF and strongly associated with advanced renal dysfunction. Both frailty and CKD independently predicted all-cause mortality and HF hospitalization, and their coexistence identified a particularly high-risk clinical phenotype. These results support the systematic integration of frailty and renal function assessments into routine HF evaluation to refine risk stratification and inform individualized therapeutic decision-making.

## 5. Conclusions

Frailty is highly prevalent among ambulatory HF patients, especially those with renal impairment. Beyond its frequency, frailty independently predicts mortality and HF hospitalization, with a stronger effect in advanced CKD. Frailty and renal dysfunction represent complementary indicators of poor prognosis in HF. Integrating both assessments into routine care may improve risk stratification and support personalized therapeutic strategies, including intensified follow-up and optimized evidence-based therapy in high-risk patients.

## Figures and Tables

**Table 1:** Baseline clinical, echocardiographic, biochemical, and treatment characteristics according to frailty status.

Variable	Total (N = 459)	Nonfrail (N = 276)	Frail (N = 183)	*p*-Value
***Baseline parameters***				
*Age*	75 [68–82]	71 [62–79]	80 [74–85]	**<0.001**
*Female Sex*	127 (27.7%)	60 (21.7%)	67 (36.6%)	**<0.001**
*Systolic blood pressure*	120 [110–130]	120 [110–130]	120 [108–130]	0.686
*Heart Rate*	70 [60–80]	70 [60–77]	71 [66–80]	**0.0013**
***Comorbidities***				
*Diabetes mellitus*	219 (47.7%)	115 (41.7%)	104 (56.8%)	**0.001**
*Hypertension*	395 (86.1%)	233 (84.4%)	162 (88.5%)	0.214
*Dyslipidemia*	262 (57.1%)	157 (56.9%)	105 (57.4%)	0.917
*COPD*	84 (18.3%)	48 (17.4%)	36 (19.7%)	0.536
*Prior Stroke*	58 (12.6%)	25 (9.1%)	33 (18.0%)	**0.005**
*Atrial fibrillation*	225 (49.0%)	114 (41.3%)	111 (60.7%)	**<0.001**
*Dialysis*	51 (11.1%)	24 (8.7%)	27 (14.8%)	**0.043**
*CKD*	362 (78.9%)	214 (73.8%)	148 (87.6%)	**<0.001**
*ISCHEMIC cardiopathy*	204 (44.4%)	117 (42.4%)	62 (33.9%)	0.067
*LVEF, %*				
*Reduced LVEF (<40%)*	206 (44.9%)	137 (49.6%)	69 (37.7%)	—
*Mildly reduced LVEF (41–49%)*	85 (18.5%)	53 (19.2%)	32 (17.5%)	—
*Preserved LVEF (≥50%)*	168 (36.6%)	86 (31.2%)	82 (44.8%)	—
	—	—	—	**0.010**
*eGFR (mL/min/1.73 m^2^)*	38.9 [24.1–63.0]	48.41 [27.9–70.3]	30.79 [20.4–45.9]	**<0.001**
***Biochemical parameters***				
*Hemoglobin (g/L)*	132 [119–146]	136 [124–148]	126 [112–141]	**<0.001**
*Serum creatinine (mg/dL)*	1.6 [1.1–2.4]	1.4 [1.0–2.1]	1.9 [1.3–2.7]	**<0.001**
*BUN (mg/dL)*	38 [25–56]	35 [23–49] (N = 240)	45 [29–68] (N = 167)	**<0.001**
*UACR (mg/g)*	36 [9–175]	25 [7–110]	58 [11–249]	**0.0008**
*Urinary sodium (mmol/L)*	63 [42–86]	66 [43–87] (N = 188)	59 [39–83] (N = 140)	0.0826
*NT-proBNP (pg/mL)*	2033 [759–5262]	1510 [523–3438]	3479 [1427–8570]	**<0.001**
*CA125 (U/mL)*	15 [9–27]	12 [8–19] (N = 229)	19 [12–43] (N = 153)	**<0.001**
*Ferritin*	151 [55–304]	132 [54–268] (N = 244)	183 [63–390] (N = 166)	**0.0139**
*TSAT*	23 [16–31]	23 [17.6–31.4] (N = 237)	22.5 [14.9–31.0] (N = 163)	0.373
***Treatment***				
*Beta-blockers*	345 (75.2%)	220 (79.7%)	125 (68.3%)	**0.006**
*Sacubitril/valsartan*	160 (34.9%)	114 (41.3%)	46 (25.1%)	**<0.001**
*ACE inhibitors/ARBs*	182 (39.7%)	112 (40.6%)	70 (38.3%)	0.618
*SGLT2i*	348 (75.8%)	209 (75.7%)	139 (76.0%)	0.955
*MRA*	190 (41.4%)	130 (47.1%)	60 (32.8%)	**0.002**
*Loop diuretics*				
*Furosemide*	228 (49.8%)	118 (42.9%)	110 (60.1%)	**<0.001**
*Torasemide*	27 (5.9%)	14 (5.1%)	13 (7.1%)	0.365
*Bumetanide*	22 (4.8%)	10 (3.6%)	12 (6.6%)	0.150
*Thiazides*	70 (15.2%)	36 (13.1%)	34 (18.6%)	0.110
*Acenocoumarol*	76 (16.6%)	49 (17.8%)	27 (14.8%)	0.397
*DOACs*	155 (33.8%)	82 (29.7%)	73 (39.9%)	**0.024**

Variables are presented separately for the overall cohort and for patients classified as nonfrail and frail. Continuous variables are expressed as median [interquartile range] and were compared between frailty groups using the Wilcoxon rank-sum test. Categorical variables are reported as counts (percentage) and were compared using the chi-square test (global χ^2^ for multi-category variables). For variables with >5% missing data, the number of available observations (n) is indicated below the corresponding values. Abbreviations: ARB, angiotensin II receptor blocker; ACEI, angiotensin-converting enzyme inhibitor; BUN, blood urea nitrogen; CA125, cancer antigen 125; CKD, chronic kidney disease; COPD, chronic obstructive pulmonary disease; DOACs, direct oral anticoagulants; eGFR, estimated glomerular filtration rate; IQR, interquartile range; LVEF, left ventricular ejection fraction; MRA, mineralocorticoid receptor antagonist; NT-proBNP, N-terminal pro-B-type natriuretic peptide; TSAT, transferrin saturation; UACR, urinary albumin-to-creatinine ratio.

**Table 2:** Univariate and Multivariate Logistic Regression for Determinants of Frailty.

Variable (Clinical Scale)	Univariate OR (95% CI)	*p*-Value	Multivariate OR (95% CI)	*p*-Value
*Age*	1.12 (1.09–1.16)	<0.001	1.12 (1.09–1.15)	<0.001
*Male sex*	0.48 (0.31–0.72)	0.001	—	—
*Diabetes mellitus*	1.84 (1.26–2.68)	0.002	—	—
*Hypertension*	1.42 (0.81–2.48)	0.216	—	—
*eGFR (mL/min/1.73 m^2^)*	0.97 (0.96–0.98)	<0.001	0.98 (0.97–0.99)	<0.001
*History of atrial fibrillation*	2.19 (1.49–3.20)	<0.001	—	—
*Prior stroke*	2.20 (1.26–3.85)	0.005	3.30 (1.66–6.57)	<0.001
*Liver disease*	2.55 (1.27–5.14)	0.008	—	—
*Jugular venous distension*	3.88 (2.14–7.03)	<0.001	—	—
*Hemoglobin*	0.97 (0.96–0.98)	<0.001	—	—
*CK (ln)*	0.36 (0.24–0.54)	<0.001	—	—
*ALP (ln)*	1.78 (1.13–2.80)	0.011	—	—
*BUN*	1.02 (1.01–1.03)	<0.001	—	—
*Albumin*	0.85 (0.80–0.90)	<0.001	—	—
*CA125 (ln)*	2.10 (1.63–2.70)	<0.001	2.18 (1.66–2.87)	<0.001
*NT-proBNP (ln)*	1.58 (1.36–1.84)	<0.001	—	—

Univariate odds ratios (ORs) with 95% confidence intervals (CIs) and *p*-values are shown for all candidate variables. All variables listed in the table were entered into a multivariate logistic regression model, and a backward stepwise selection procedure was applied. The final multivariate model retained age, eGFR (mL/min/1.73 m^2^), prior stroke, and CA125 (natural logarithm). Frailty status was the dependent variable, with nonfrail patients serving as the reference category. Biomarkers with skewed distributions were log-transformed (LN). No imputation of missing data was performed. Abbreviations: OR, odds ratio; CI, confidence interval; ALP, alkaline phosphatase; BUN, blood urea nitrogen; CA125, cancer antigen 125; CK, creatine kinase; eGFR, estimated glomerular filtration rate; NT-proBNP, N-terminal pro–B-type natriuretic peptide; LN, natural logarithm.

**Table 3:** Univariate and Multivariate Cox Regression Analyses for the Composite Outcome of All-Cause Death or Heart Failure Hospitalization at 1 Year.

Variable (Clinical Scale)	Univariate HR (95% CI)	*p*-Value	Multivariate HR (95% CI)	*p*-Value
*Frailty*	2.81 (1.77–4.43)	<0.001	2.09 (1.22–3.58)	0.007
*Age*	1.02 (1.00–1.05)	0.028	—	—
*eGFR (mL/min/1.73 m^2^)*	0.98 (0.97–0.99)	0.004	—	—
*History of atrial fibrillation*	2.07 (1.29–3.32)	0.002	—	—
*Dialysis*	1.87 (1.02-3.43)	0.040	—	—
*Jugular venous distension*	3.49 (2.08–5.84)	<0.001	—	—
*CK (ln)*	0.55 (0.34–0.90)	0.017	—	—
*BUN*	1.00 (1.00–1.01)	0.007	—	—
*Albumin*	0.88 (0.84–0.93)	<0.001	—	—
*CA125 (ln)*	1.69 (1.38–2.07)	<0.001	1.31 (1.10–1.62)	0.003
*NT-proBNP (ln)*	1.59 (1.36–1.86)	<0.001	1.33 (1.04–1.66)	0.022

Hazard ratios (HRs) with 95% confidence intervals (CIs) and *p*-values are presented for the association of each clinical and biochemical variable with the primary outcome. Variables with *p* < 0.10 in univariate analyses, together with clinically relevant covariates, were entered into a multivariate Cox model using backward stepwise selection. The final adjusted model retained frailty, CA125 (log-transformed), and NT-proBNP (log-transformed) as independent predictors. Log transformation (LN) was applied to biomarkers with skewed distributions to meet model assumptions. Abbreviations: HR, hazard ratio; CI, confidence interval; BUN, blood urea nitrogen; CK, creatine kinase; eGFR, estimated glomerular filtration rate; CA125, cancer antigen 125; NT-proBNP, N-terminal pro–B-type natriuretic peptide; LN, natural logarithm.

## Data Availability

The data supporting the findings of this study are available from the corresponding author upon reasonable request. Individual patient-level data cannot be made publicly available due to institutional and ethical restrictions.

## Supplementary Materials

The following supporting information can be downloaded at: https://www.mdpi.com/article/10.3390/life16010045/s1, Table S1: Prevalence of Frailty Domains in Frail and Non-Frail Patients.
