# Supplementary material for: Interaction Between Frailty and Renal Function in Patients with Heart Failure
Source: Life (Basel). 2025 Dec 26;16(1):45. doi: 10.3390/life16010045 (PMC12842626; doi:10.3390/life16010045)
Supplement: Supplementary file 1 [file life-16-00045-s001.zip › life-4017836-supplementary.pdf]

Supplementary Table S1. Prevalence of Frailty Domains in Frail and Nonfrail Patients.

	FRAIL (N=183)	NONFRAIL (N=276)	TOTAL (N=459)
Unintentional weight loss	106 (57.9%)	20 (7.3%)	126 (27.5%)
Weakness	175 (95.6%)	107 (38.8%)	282 (61.4%)
Exhaustion	161 (88.0%)	93 (33.7%)	254 (55.3%)
Slowness	91 (49.7%)	6 (2.2%)	97 (21.1%)
Low physical activity	165 (90.2%)	49 (17.8%)	214 (46.6%)

Absolute numbers and corresponding percentages within each frailty group are shown. Domains are based on Fried's physical frailty phenotype: uninten onal weight loss, weakness, exhaus on, slowness, and low physical ac vity. Comparisons between groups were performed using the chi-square test. Abbrevia on: n = number of patients.
